# Brighton, and Its Three Climates; Remarks on Its Medical Topography, and Advice and Warnings to Invalids and Visitors

**Published:** 1843-10

**Authors:** 


### Art. XIX.

*Brighton, and its three Climates; Remarks on its Medical Topography, and Advice and Warnings to Invalids and Visitors.* By A. L. Wigan, m.d. Surgeon, formerly practising in that Town. — *London*, 1842. 8vo, pp. 72.

This is a pretty little pamphlet, neatly and smartly written, and will be found very useful to the thousands who visit Brighton for the purpose of deriving benefit from its pure and bracing climate. It briefly points out a good many little things which invalids ought to seek and shun. It, however, hardly fulfils its promise, inasmuch as it gives us extremely little information as to the *climates* so ostentatiously set forth in the title-page. The following extract contains nearly every word said on this subject, and though the statements are accurate, as far as they go, they scarcely announce such differences as suffice to constitute variety of climate. We believe the three divisions of the town really to possess, respectively, qualities capable of influencing the health of delicate persons; and in recommending our patients to Brighton, in common with other physicians, we have been in the habit of indicating their residence accordingly. But we have to complain that Dr. Wigan rather indicates the localities that describes the climates.

"The town of Brighton may be conveniently divided into three parts: the centre portion, extending from Cannon Place on the west, to the New Steyne, or even to Rock Gardens, on the east; and the two extremities, to Adelaide Terrace on the west, and to Arundel Terrace, Kemp Town, on the east, each inclusive.

"Although this division is arbitrary, and the characteristics of each portion fade off insensibly into each other, it is sufficiently distinct for my purpose, and I shall use the terms *Eastern*, *Western*, and *Centre* portion when speaking of the influence of locality on disease, and recommend or forbid them accordingly.

"I. The Centre portion, being occupied by almost all the permanent inhabitants of the town, is necessarily the most frequented; and as all these persons have connexions in London, is, of course, most frequently recommended to strangers. It differs, however, very little from any inland town placed in a low situation, and it possesses none of the quality commonly called 'bracing.' Much annoyance is occasioned by the numerous steam-engines belonging to the baths and breweries, and the chimneys of private houses in the Western and Eastern portions of the town, according to the direction of the wind, which blows from those quarters respectively about eight months and two months throughout the year—north and south for the remainder.

"When the wind is due north the atmosphere at all seasons is beautifully transparent, and the whole town as brilliant as Genoa. In ordinary weather, although each extremity of the town is extremely clear, the middle portion has a mist (at least) which prevents the free ascent of the smoke, and the inhabitants of one end of the town can scarcely ever see the other extremity—each generally supposing the other end to be enveloped in smoke, when, in fact, it is as clear as his own portion.

"The Centre of the town has been so effectually drained, and there is so much care employed to remove nuisances, that it is not objectionable to those in health, nor, indeed, to more than one class of invalids, and it presents the advantage of baths, libraries, shops, riding-schools, fencing-rooms, billiards, and other sources of health or recreation.

"To individuals without families, and who are unable to have a permanent conveyance, and to those who resort to the baths for maladies requiring surgical aid only, or who merely come to Brighton to escape the turmoil of business, or the thick atmosphere of London, this part of the town is well adapted; but such as have their families with them, and can afford the larger and better-built houses of the East and West, should not subject themselves to the disadvantages and annoyances inseparable from a low situation in the centre of a large town.

"II. The Eastern portion of Brighton is elevated considerably above the sea, and, except when the wind is violent, escapes in a great degree the admixture of saline particles with the atmosphere; not chemically, but mechanically suspended therein. It has a chalky soil, through which the rain immediately percolates, and which permits no moisture to remain on the surface. The houses, too, are of modern erection, and possess many of the comforts and conveniences which have only lately been brought into general use; and the air is decidedly 'bracing.'

"III. The Western portion, again, (at least that part of it which abuts on the sea,) has a clayey soil, and a mild and soft climate. The clay has, however, been so largely excavated for building and for bricks, and the immense lawn in front of the sea is so entirely composed of artificial soil, covering a bed of rounded pebbles, and the country further to the west in the direction of the prevalent winds is so entirely gravel, that it escapes in a great degree the annoyances which the original composition of the soil would have seemed to indicate. I consider that portion of the Western division of the town which is north (and, perhaps, inclusive) of the Western Road; the upper part of Montpellier Road; extending to the New Vicarage, and the house called the Temple, and round to the Poor House, to be by far the most salubrious portion of the whole town There are indeed very few, if any, maladies for which the air of Brighton is advisable that will not be more favorably placed here than elsewhere." (pp. 25-9.)

As Dr. Wigan has resided many years at Brighton, and is evidently a shrewd and a good observer; and as no suspicion can attach to his testimony on the score of an interested partiality, (he having left the place,) we regard the few following remarks, gleaned from various parts of his pamphlet, as well meriting the attention of all who send or are sent to Brighton.

"It is of the very essence of atmospheric influence, that if it benefit one class of diseases, it must necessarily aggravate those of an opposite character, and the air of Brighton, as far as his experience extends, is never neutral." (p. 14.)

"There is no proposition of which I am more firmly convinced, than that, in all cases whatever, the sea-air is more salubrious at the distance of five or six hundred yards from the edge of the water than close to it." (p. 30.)

"From the middle of March to the middle of May, unless in compliance with deliberate medical advice by one who thoroughly understands the subject, and to whom you have given ample opportunities of knowing your disease, stay away altogether. I know no malady whatever, not even glandular disease, which is benefited by sea-air thus early in the season." (pp. 32-3.)

"From the middle of July to the middle of October, invalids may locate themselves as close to the water as they please, or on it. At other seasons let them keep at a short distance, and if they have the power to choose, select a house with an eastern aspect. This, by the by, from the structure of the town, will generally be south-eastern; take it altogether, the best 'exposition' of all." (p. 32.)

"The month of June, when (as the phrase is) there is 'not a soul in Brighton,' which, as in London, means souls and bodies of a certain class; this month and the next are not merely the most delightful period of the year, but that in which the air of the sea at Brighton has the most energetic influence in the cure of disease. The sea-fogs are over; the air is become of an uniform temperature, not too warm to admit of abundant exercise; the pathways and roads through the corn-fields afford delightful walks and rides; the Downs are in perfection; open carriages may be had on exceedingly moderate terms." (pp. 34-5.)

"The Downs, which surround the town, furnish the purest and most exhilarating air in the world. Never on the Alps, the Appenines, or the Jura have I felt so intensely, so exultingly, the abstract pleasure of mere animal existence, as on the Downs in the neighbourhood of Brighton. No manure, no decomposition on the surface, because no humidity will remain there; at such a distance, or such an elevation above the sea, that all which is insalubrious in the air has been deposited before it reaches them. A canter over the Downs in a fine day produces the feelings of the Arab in the desert: the breathing deep and complete, and every air-cell of the lungs fully opened and performing its duty." (pp. 39-40.)

"Speaking generally, and with exceedingly rare exceptions, (if any,) all congestive diseases are aggravated by a residence at Brighton. By 'congestive,' I mean such diseases as are caused or accompanied by local accumulation of blood, principally venous, circulating languidly in any of the organs of the body, and unattended with inflammation." (pp. 40-1.)

"Inflammatory dyspepsia is another of the ailments for which Brighton is objectionable; and, generally, all the diseases of the kidneys and bladder which are not attended with a considerable secretion of albumen, the latter being apparently benefited by the atmosphere which aggravates the others. Such at least is the result of my own experience in these obscure diseases." (p. 42.)

"Many invalids, whose ailments were stationary or retrograde while residing in face of the sea, or very near to it, have recovered immediately when I have sent them to the top of Montpellier Road, Montpellier Terrace, to the New North Road; to the row of cottages near the Poor House called Vine Place, or even to the Western Road, especially the western part of it; but these places afford few good houses." (pp. 43-4.)

"That delightful promenade, the Chain Pier, is so frequent a cause of indisposition in the early part of the year, that I have been accustomed to say that it ought to be maintained at the expense of the medical men of the town, from the practice it brings them. The sheltered walk under the cliff (which leads to it) affords a delightful resource, when a bitter north-eastern makes other places disagreeable. The perfect defence from the wind, with the benefit of a winter or spring sun reflected from the Cliffs, gives quite the feeling of summer, and this degree of shelter and warmth will extend perhaps to half the length of the Pier itself. At a certain point the protection of the Cliff ceases; you pass from a calm air (under the Cliff) at fifty-five or sixty degrees, to a keen wind at thirty-five [?] or forty, which from its rapidity produces the effect of a frost. The bright day has perhaps induced some change in the clothing, and with women and children especially, the mischief is often done in a few minutes." (pp. 45-6.)

"A common objection to Brighton on the part of Londoners is, that it is injurious to the eyes. This opinion, no doubt, takes its rise from the pain which often accompanies the comparatively permanent contraction of the pupil on changing the moderate light of a town for the vivid light of the south coast. It is a pain which ceases at the end of a few days, and, as far as I recollect, is always followed by an improvement of the vision. I know no exception. Brighton has the most extraordinary exemption from disease of the eyes of any place in Europe that is known to me." (p. 47.)

We wish Dr. Wigan would write a completer work on Brighton, pointing out the precise nature and peculiarities of its climate or climates, and its or their effects on different constitutions, temperaments, and diseases. This little work proves him to be well qualified for the task.

